# Gangrenous gas necrosis of the spleen: a case report

**DOI:** 10.1186/s12879-019-4406-4

**Published:** 2019-09-05

**Authors:** Jeremy Meyer, Arnaud Dupuis, Benedikt D. Huttner, Matthieu Tihy, Léo Bühler

**Affiliations:** 1 Division of Digestive Surgery, University Hospitals of Geneva, Rue Gabrielle-Perret-Gentil 4, 1211 Genève 14, Switzerland; 2 Division of Infectious Diseases, University Hospitals of Geneva, Rue Gabrielle-Perret-Gentil 4, 1211 Genève 14, Switzerland; 3 Division of Pathology, University Hospitals of Geneva, Rue Gabrielle-Perret-Gentil 4, 1211 Genève 14, Switzerland

**Keywords:** Splenic abscess, Splenic gangrene, Spleen, Gangrene, Spontaneous gas gangrene

## Abstract

**Background:**

Splenic abscess usually arises from hematogenous spread. Causative pathogens are various and anaerobic pathogens are rarely reported.

**Case presentation:**

We report the case of a 50-year-old male patient who was admitted for sepsis due to gangrenous necrosis of the spleen associated with bacteremia. Causative pathogens were *Clostridium perfringens* and *Streptococcus gallolyticus*. The patient was successfully treated by splenectomy and targeted intravenous antibiotics. No underlying or predisposing disease was found.

**Conclusion:**

Gangrenous necrosis of the spleen is a rare entity that can be successfully treated by splenectomy and antibiotics.

## Background

Splenic abscess constitutes an uncommon entity, usually arising from hematogenous spread [1, 2]. Numerous different causative pathogens have been reported in the literature such as *Escherichia coli*, *Proteus mirabilis*, *Streptococcus* spp., *Klebsellia pneumoniae*, *Staphylococcus aureus*, *Salmonella* spp., *Enterococcus* spp., *Pseudomonas* spp. [2–6]. Predominant anaerobic pathogens were reported to be *Peptostreptococcus* spp., *Bacteroides* spp., *Fusobacterium* spp., *Clostridium* spp. and *Propionibacterium acnes* [4, 7]. Splenic abscess of fungal origin may arise in immunocompromised patients, such as those with HIV or hematological cancer [3]. Diagnosis is usually made by ultrasonography or computed tomography. Treatment relies either on percutaneous drainage [8, 9] or surgery. However, due to the high failure rate of percuteanous drainage, surgery (splenectomy) often constitutes the definitive treatment for splenic abscesses.

Here, we report the rare case of a spontaneous splenic abscess due to *Clostridium perfringens* and *Streptococcus gallolyticus* in an immunocompetent patient.

## Case presentation

**Fig. 1:**
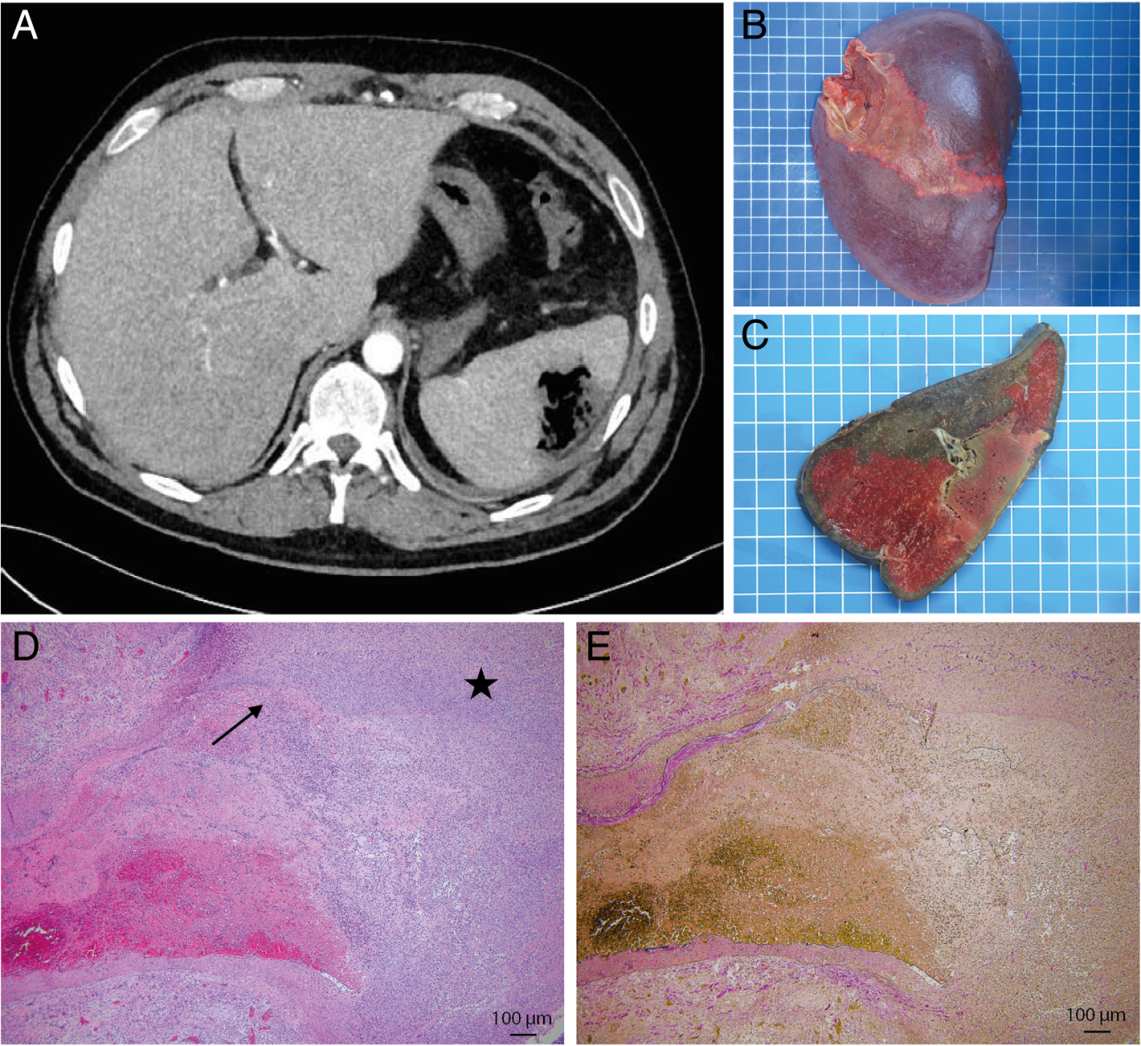
**a** Computed tomography axial picture showing the necrotic splenic abscess, **b** Operative specimen (spleen), **c** Sagittal section of the operative specimen (spleen), **d**-**e** Photomicrographs of the aneurysm of the splenic artery (D. H&E staining and E. Miller’s Elastin Staining) showing destruction of the tunica media (arrow) surrounded by splenic infarction (star)

We report the case of a 50 year old male patient, known for a ischemic cerebral ischemia due to a foramen ovale in 1997, closure of that foramen in 2010, who was complaining of left flank abdominal pain and fever since 4 days. He went to a private clinic twice and was discharged with symptomatic treatment and without antibiotics. The patient had no history of trauma. At physical examination, we noted the presence of a fever at 38.6 °C, tachycardia at 100 bpm but normal blood pressure. The left flank was sensitive at palpation without rebound pain. Blood tests indicated acute inflammation, with a leukocytosis at 17.2 G/l and a CRP value at 337 mg/l. Hemoglobin, hemostasis parameters, creatinine, liver and pancreatic tests were within the normal ranges. Arterial blood pH and lactate values were also normal. Computed tomography showed a necrosis of the spleen with air, associated with a thin ipsilateral pleural effusion (Fig. 1a). The patient received 3 l of cristalloids, ceftriaxone and metrodinazole and was transferred to intensive care unit for monitoring. Blood cultures were positive for *Clostridium perfringens* and *Streptococcus gallolyticus*. Antibiotics were switched to amoxicilline-clavulamate and clindamycin, the latter for its potential effect on clostridial toxin production through interference with ribosomal protein synthesis [10]. A transthoracic cardiac utrasonography was performed and showed a normal cardiac function without valvular dysfunction nor endocarditis. After 12 h, we performed a splenectomy by midline incision. Samples of perisplenic purulent liquid were harvested. The spleen was exposed, allowing visualizing a voluminous perforated abscess (Fig. 1b-c). Short vessels were sectioned using a sealing device and main vessels were controlled using non-absorbable ligatures, staying at distance from the pancreas. The spleen was extracted. After local washing, a drain was left in place, the abdominal wall was closed using absorbable stiches and a prophylactic cutaneous negative therapy device was applied. Perioperative bacteriological samples were positive for *Clostridium perfringens, Streptococcus gallolyticus* and *Clostridium baratii*. The patient stayed 24 h in perioperative care unit before returning to the ward. The drain was removed after 6 days and antibiotics were continued for 14 days. Endocarditis was definitively excluded using transoesophageal ultrasonography. HIV serology was negative. To rule out associated pathology of the gastro-intestinal tract, a colonoscopy was performed and was normal. The patient was vaccinated against *Streptococcus pneumonia, Neisseria meningitidis* and *Influenza*, before being discharged 17 days after admission. Microscopic pathologic examination showed also a ruptured aneurysm (Fig. 1d-e) of a branch of the splenic artery, surrounded by a splenic infarction.

## Discussion

We report the case of a splenic abscess of polymicrobial origin, due to *Clostridium spp* and *Streptococcus gallolyticus with gas gangrene*. The patient was successfully managed by splenectomy and 2 weeks of targeted antibiotic treatment. *Clostridium perfringens* and *Streptococcus gallolyticus* were identified from blood cultures, whereas another pathogen, *Clostridium baratii* was retrieved from the perisplenic liquid. Anaerobic splenic abscesses due to *Clostridium perfringens* are seldom reported in the literature [4, 7, 11, 12]. In most cases, a predisposing factor exists, as immunodeficiency, hematologic disease, trauma or infectious [12, 13]. Further, spontaneous gas gangrene is mostly due to *Clostridium septicum* and cases due to *Clostridium perfringens* are rarely reported [13–15]. Splenic aneurysm is also a rare complication of infectious diseases, trauma or disorders of the haematopoietic system but could also occur spontaneously [16–19].

In our case, the patient was immunocompetent, did not have hematologic disease, evidence for endocarditis nor history of trauma whose origin we assume was probably hematogenic.

Bacteriemia due to *Clostridium* spp. and especially *Streptococcus gallolyticus* is reported to be associated with gastrointestinal diseases, especially colorectal cancer [16]. Therefore, we performed a colonoscopy, which did not show any signs of colonic neoplasia. Also, bacterial translocation following for example an episode of gastroenteritis is unlikely, as the patient had no diarrhea or vomiting.

In conclusion, we report the case of a splenic abscess of polymicrobial origin, due to *Clostridium* spp. and *Streptococcus gallolyticus* with gas gangrene, in an immunocompetent patient without any evidence for endocarditis or trauma. The patient was successfully treated by splenectomy and antibiotics and could be discharged 17 days after admission. Attention should be given to exclude endocarditis, immunosuppression and colonic diseases in such patients.

## Data Availability

n/a.
